# Poly(ε-Caprolactone) Hollow Fiber Membranes for the Biofabrication of a Vascularized Human Liver Tissue

**DOI:** 10.3390/membranes10060112

**Published:** 2020-05-27

**Authors:** Simona Salerno, Franco Tasselli, Enrico Drioli, Loredana De Bartolo

**Affiliations:** 1 CNR-ITM, Institute on Membrane Technology, National Research Council of Italy, via P. Bucci, cubo 17/C, I-87036 Rende, Italy; s.salerno@itm.cnr.it (S.S.); f.tasselli@itm.cnr.it (F.T.); e.drioli@itm.cnr.it (E.D.); 2 Department of Energy, Engineering, Hanyang University, Seoul 04763, Korea

**Keywords:** poly(ε-caprolactone) hollow fiber membranes, membrane bioreactor, bioartificial organs, vascularized hepatic tissue, liver specific functions

## Abstract

The creation of a liver tissue that recapitulates the micro-architecture and functional complexity of a human organ is still one of the main challenges of liver tissue engineering. Here we report on the development of a 3D vascularized hepatic tissue based on biodegradable hollow fiber (HF) membranes of poly(ε-caprolactone) (PCL) that compartmentalize human hepatocytes on the external surface and between the fibers, and endothelial cells into the fiber lumen. To this purpose, PCL HF membranes were prepared by a dry-jet wet phase inversion spinning technique tailoring the operational parameters in order to obtain fibers with suitable properties. After characterization, the fibers were applied to generate a human vascularized hepatic unit by loading endothelial cells in their inner surface and hepatocytes on the external surface. The unit was connected to a perfusion system, and the morpho-functional behavior was evaluated. The results demonstrated the large integration of endothelial cells with the internal surface of individual PCL fibers forming vascular-like structures, and hepatocytes covered completely the external surface and the space between fibers. The perfused 3D hepatic unit retained its functional activity at high levels up to 18 days. This bottom-up tissue engineering approach represents a rational strategy to create relatively 3D vascularized tissues and organs.

## 1. Introduction

The creation of a liver tissue that recapitulates the micro-architecture and functional complexity of a human organ is still one of the main challenges of liver tissue engineering. Many efforts are devoted to fabricate bioengineered livers with a dual purpose to create in vitro tools for xenobiotics testing, toxicological studies and as disease models, and on the other hand to develop bioartificial liver systems to sustain liver patient’s lives before liver transplantation [1,2,3,4]. Significant progresses have been experienced in the area of cell engineering, biomaterials fabrication, and tissue architecture accomplishing important achievements in terms of phenotypic and specific hepatic functions. Over the years, a number of strategies have been identified to recapitulate aspects of the in vivo microenvironment for the rescue of hepatic phenotype in culture. Hepatocyte viability and functions can be enhanced or maintained by improving the cell culture microenvironment (e.g., cell–cell and cell–matrix interactions, surface modification), implementing heterotypic co-culture models and by using 3D models such as collagen sandwich, 3D cell printed constructs and spheroids [5,6,7,8]. In addition, perfusion-based approaches as new 3D culture systems have emerged and showed better potential to model in vivo tissue microenvironment state. In spite of conventional 2D monolayer systems, human liver cells under perfusion conditions such as in a membrane bioreactor maintained their specific functions at high levels for about 1 month, leading to the formation of highly organized microtissues that mimic the native liver tissue [9,10,11]. The use of membranes and membrane devices is of key interest in the development of bioartificial organs and tissues given the large number of configuration (flat, hollow fiber, interwoven, microtube array, multibore) that can be created. Membranes can emulate the essential characteristics of the physiological environment, including tissue-specific extracellular membrane (ECM) interactions serving as substrate for cell adhesion, 3D-microarchitecture, and stiffness, selective transport of nutrients to cells and removal of catabolites from cell compartment in order to support cellular growth and maintain liver cell phenotypes [4].

Scientific reports highlighted an enhanced function of liver cells in co-culture with non-parenchymal cells or mesenchymal cells under 2D and 3D culture models in membrane bioreactors, which have been shown to provide an adequate cell oxygenation [12,13,14]. In these devices, hepatocytes together with non-parenchymal cells were mainly cultured over the external surface of commercial non-biodegradable hollow fiber membranes assembled in parallel, in a network or in a crossed configuration. These studies have demonstrated that membrane platforms are able to reproduce in vitro the physiological behavior of liver cells.

Taking into account the previous achievements, here we report on the development of a 3D vascularized human hepatic tissue based on biodegradable hollow fiber (HF) membranes of poly(ε-caprolactone) (PCL) that compartmentalize human hepatocytes on the external surface and endothelial cells into the lumen of the fibers. In such configuration, endothelial cells colonize the lumen of the fibers forming vascularized channels, and communicate with hepatocytes present in the extraluminal compartment through their secreted molecules permeating across the microporous structure of the membrane wall. The approach consisted in the synthesis of hollow fiber membranes by using PCL that is aliphatic, biocompatible and biodegradable polyester approved by FDA. It is an interesting material with a wide processing range thanks to its low melting temperature (*T*_m_ = 60 °C) and high decomposition temperature (*T*_d_ = 350 °C), which enables the fabrication of a variety of structures and forms [15]. To this purpose, PCL HF membranes were prepared by a dry-jet wet phase inversion spinning technique tailoring the operational parameters in order to obtain PCL fibers with suitable properties. Then, the fibers were characterized in order to establish their structural, physico-chemical, mechanical and permeable properties, which play a critical role in the cell adhesion and growth. The rational design involved the formation of a human hepatic unit by packing the fiber bundle in a housing to establish the intraluminal and extraluminal compartments where human endothelial cells and hepatocytes were cultured, respectively, under perfusion conditions. This combination likely helps to generate a human vascularized hepatic unit by using physiologically relevant cells such as human endothelial cells that constitute the inner surface of the fiber as in the in vivo vasculature, and hepatocytes that adhered on the external surface and between fibers. The morphological and functional behavior of the hepatic construct was evaluated by assessing the liver specific functions.

## 2. Materials and Methods

### 2.1. Membrane Preparation and Characterization

PCL HF membranes were prepared according to the dry-jet wet spinning method, using a polymeric solution of PCL with high MW (Mn~70,000–90,000 by GPC, Sigma-Aldrich, Milan, Italy) 15 wt%, 1-methyl-2-pyrrolidinone (NMP) 75 wt%, and glycerol 10 wt%, obtained under continuous mechanical stirring at 70 °C. For the spinning process we used a pilot implant constituted of several parts, of which the main ones were purchased from 3V Mabo Spa, Fiorenzuola d’Arda, Italy. The polymeric or dope solution was pumped through the spinneret with flow rate of 9 g/min. The bore injection fluid, consisting of ultrapure water, was simultaneously pumped through the inner tube of the spinneret with flow rate of 6 g/min. After a short residence time in the air (air gap), the fiber was immersed in the non-solvent bath constituted of water at 20 °C, where coagulation occurred. The dope solution was kept at 20 °C in the syringe during spinning and extruded through a spinneret with outer and inner needle diameters of 2 mm and 1 mm, respectively. The distance between the spinneret and the coagulation bath, also known as the air gap, was 30 cm and the take up speed was 4.8 m/min. Finally, the prepared membranes were stored in distilled water for more than 24 h to remove the residual solvents. After washing, the HFs were characterized and used for cell culture.

The structural properties of the PCL HF membranes were characterized by scanning electron microscopy (SEM). Surface and cross-section samples were mounted with double-faced conductive adhesive tape and analyzed by SEM (ESEM FEG QUANTA 200, FEI Co., Hillsboro, OR, USA). SEM images allowed to investigate the morphological characteristics of the fibers. The cross-section, wall thickness, inner and external diameter as well as pore sizes of the membranes were measured on digital images by using NIH Image.

For the characterization of hydraulic permeability properties, the PCL HF membranes were potted inside glass modules (with length and inner diameter of 20 cm and 1.5 cm, respectively) to access to the intraluminal and extraluminal compartments. Ultrapure water was circulated through a peristaltic pump from the reservoir into the inlet port of the module while monitoring continuously pressures at inlet and outlet of the module by online manometers (Allemano, Grugliasco (TO), Italy). Measurements of extraluminal flow (permeate) were performed at different transmembrane pressures (**Δ**P^™^) ranging from 0 to 120 mbar, which were obtained by varying inlet pressures. The flux through the membranes (J, in L/m^2^·h) was plotted as a function of the **Δ**P^™^, in mbar and the permeance (Lp, in L/m^2^·h·mbar) was calculated from the slope of this curve assuming a linear correlation between water flux and the convection driving force. The transmembrane flux vs. **Δ**P^™^ was evaluated on four modules and the average values of water permeability were reported. After evaluation of the hydraulic permeance, the solute permeation measurements were performed by using glucose, albumin and apotransferrin (Sigma-Aldrich, Milan, Italy). Test solutions were prepared by dissolving separately 0.5 mg/mL of each metabolite in phosphate buffer at pH 7.4 and the permeate flux was continuously measured. The concentration of metabolites permeating through the membranes was monitored and assessed by an online UV-spectrophotometer (LKB Uvicord SII, Pharmacia, East Lyme, CT, USA) except for glucose, which was determined by phenol assay.

Mechanical properties of the PCL HF membranes, in terms of Tensile Modulus E, Ultimate Tensile Strength and elongation at break ε, were estimated via tensile test (Zwich/Roell Z2.5 machine, Ulm, Germany) on 10 samples with length of 5 cm. The testXpert**^®^** testing software was used for the analysis of the acquired real-time longitudinal deformation measurements. Tensile tests were carried out at 20 °C applying a pre-load of 0.05 MPa and a speed of 4 mm/min on samples in dry and wet conditions. Wet samples were analyzed after an incubation of 6 h in PBS buffer.

### 2.2. Membrane Bioreactor and Fluid Dynamics Characterization

**Figure 1:**
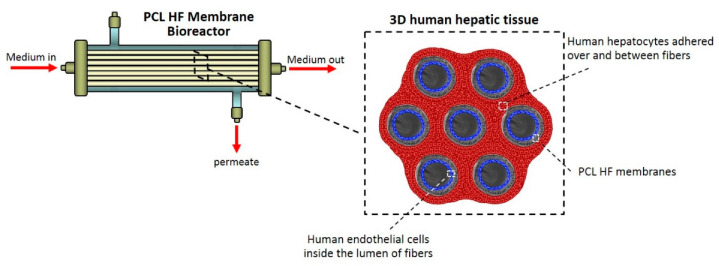
Poly(ε-caprolactone) (PCL) hollow fiber (HF) membrane bioreactor and scheme of the 3D human hepatic tissue realized by culturing human hepatocytes over and between PCL HF membranes parallel assembled at a distance of 250 µm, and endothelial cells compartmentalized in the lumen of the fibers. The cells were in communication through the porous wall of the membranes.

The membrane bioreactor consists of 7 PCL HF membranes located in parallel at a distance of 250 μm each other, inside a glass housing (length: 13 cm, inner diameter: 1.5 cm) and potted at each end, in order to establish two separate compartments: an extraluminal compartment or shell outside the fibers, and an intraluminal one within the fibers. The two compartments communicate through the pores in the fibers wall (Figure 1). PCL HF membranes inside the bioreactor provided an outside and inner surface areas of 40 cm^2^ and 27.5 cm^2^, respectively.

The dynamic culture was achieved connecting the bioreactor to a perfusion system composed of a tubing circuit, medium reservoir and waste, and micro peristaltic pumps that ensured a continuous feeding of fresh medium in the intraluminal compartment, and the collection of the same from the extraluminal compartment. A feeding flow rate (Q_in_) of 0.5 mL/min was established taking into account fluid dynamics characterization and optimization. Media samples were daily collected from the PCL HF bioreactor to evaluate the cell viability and functions. A single-pass perfusion was applied for the feeding of fresh medium that was collected as waste stream leaving the bioreactor, Q_out_, until the steady state was reached. After that, the stream leaving the bioreactor was recycled in order to obtain the accumulation of products. The bioreactor fluid dynamics was characterized without cells in terms of Residence Time Distribution by tracer technique.

### 2.3. Cell Cultures

Cryopreserved primary human hepatocytes (Gibco^TM^, ThermoFisher Scientific, Rodano (MI), Italy) isolated from non-transplantable tissue of young single donors, and Human Umbilical Vein Endothelial Cells (Cascade Biologics, Mansfield, UK) were used for cell culture experiments and the creation of a vascularized hepatic unit.

Primary Human Umbilical Vein Endothelial Cells were previously expanded in culture Medium 200 (Cascade Biologics, Mansfield, UK) containing the Low Serum Growth Supplement kit, LSGS kit (Cascade Biologics, Mansfield, UK) constituted of 1 µg/mL hydrocortisone, 10 ng/mL human epidermal growth factor (hEGF), 3 ng/mL basic fibroblast growth factor (bFGF), 10 µg/mL heparin, 10 µg/mL gentamicin, 0.25 µg/mL amphotericin B and 2% fetal bovine serum (FBS) [14]. Endothelial cells with 4 population-doubling levels were seeded at a cell density of 1.25 × 10^4^ cell/cm^2^ in the lumen of PCL HF membranes previously conditioned with Medium 200 supplemented with LSGS kit and 2% FBS, and incubated at 37 °C in a 5% CO_2_; 20% O_2_ atmosphere (*v*/*v*) with 95% relative humidity.

After 4 h, primary human hepatocytes were seeded on the external surface of the PCL HF membranes at a cell density of 1.25 × 10^5^ cell/cm^2^. Cell densities were defined on the basis of the optimized 10:1 for human hepatocytes and endothelial cells [14]. Primary human hepatocytes were previously thawed and then re-suspended in Williams’ Medium E supplemented with all the components provided by Cocktail A plating supplements (ThermoFisher Scientific): 1 µM dexamethasone, 100 U/mL penicillin, 100 µg/mL streptomycin, 4 µg/mL human recombinant insulin, 2 mM GlutaMAX™, 15 mM HEPES pH 7.4, and 5% fetal calf serum (FCS), and centrifuged at 50 g at 4 °C for 5 min. The cell pellet was tested for the cell viability by Trypan blue exclusion. Cells were incubated at 37 °C in a 5% CO_2_; 20% O_2_ atmosphere (*v*/*v*) with 95% relative humidity, in static conditions for 24 h in a culture medium consisting of 1:1 of William’s Medium E plus Cocktail A/Medium 200 plus LSGS kit, and with 2% FCS. Thereafter the membrane bioreactor was connected to the perfusion system and maintained in dynamic condition in a media constituted of 1:1 of William’s Medium E plus Cocktail B/Medium 200 plus LSGS kit, and with 1% FCS and Diazepam 10 µg/mL, for the whole culture time. Cocktail B is a mixture of cell maintenance supplements (ThermoFisher Scientific, Rodano (MI), Italy) constituted of: 0.1 µM dexamethasone, 50 U/mL penicillin, 50 μg/mL streptomycin, 6.25 µg/mL human recombinant insulin, 6.25 µg/mL human transferrin, 6.25 ng/mL selenous acid, 5.35 µg/mL linoleic acid, 2 mM GlutaMAX™, 15 mM HEPES pH 7.4.

Homotypic cultures of endothelial cells were obtained seeding the cells with 4 population–doubling levels at a density of 1.25 × 10^4^ cell/cm^2^ in the lumen of PCL HF membranes in Medium 200 supplemented with LSGS kit and 1% FCS. Homotypic cultures of liver cells were realized seeding primary human hepatocytes at a density of 1.25 × 10^5^ cell/cm^2^ on the external surface of the PCL HF membranes, and maintaining cells in William’s Medium E plus Cocktail B with 1% FCS and Diazepam 10 µg/mL. Both the homotypic cultures were used as controls in different PCL HF membrane bioreactors, respectively, connected to perfusion systems in dynamic conditions for the whole culture time.

### 2.4. Cell Morphology

Morphology of primary human endothelial cells and primary human hepatocytes cultured in the lumen and on the external surface of the PCL HF membranes, respectively, was evaluated by SEM and Confocal Laser Scanning Microscopy (CLSM, Fluoview FV300, Olympus, Segrate (MI), Italy) analysis after 18 days of culture in the bioreactor under dynamic conditions.

For SEM analysis, cell cultures specimens were previously fixed for 30 min in 2.5% glutaraldehyde, pH 7.4 phosphate buffer, and for further 30 min in 1% osmium tetroxide, and thereafter progressively dehydrated in ethanol solutions.

For CLSM investigation, specific cellular markers of cell cultures specimens were properly immunostained. Samples collected from the PCL HF bioreactor after 18 days of culture were washed with PBS, fixed for 15 min in 3% paraformaldehyde, permeabilized for 5 min in 0.5% Triton-X100, and saturated with 5% normal donkey serum (NDS), as previously described [14]. Actin was stained with phalloidin-Alexa 488 conjugated (Molecular Probes, Inc., Eugene, OR, USA) incubated for 30 min. The hepatic bile duct marker cytokeratin CK19 was visualized with a goat monoclonal antibody anti-human CK19 (Santa Cruz Biotechnology, Santa Cruz, CA, USA) and a Cy**^™^**5-conjugated AffiniPure donkey anti-goat IgG (Jackson ImmunoResearch Europe Ltd., Cambridge, UK). Endothelial cells were visualized for the glycoprotein CD31, using a mouse monoclonal antibody raised against the glycoprotein CD31 of human origin (BD Biosciences, Franklin Lakes, NJ, USA) and a Cy**^™^**3-conjugated AffiniPure donkey anti-mouse IgG (Jackson ImmunoResearch Europe Ltd., Cambridge, UK). All primary and secondary antibody were incubated for 2 and 1.5 h RT, respectively. Counterstaining for nuclei was performed with DAPI 0.2 μg/mL (Molecular Probes Inc, Eugene, OR, USA) incubated for 30 min. Finally, samples were rinsed, mounted, and viewed with CLSM (Fluoview FV300, Olympus).

### 2.5. Biochemical Assays

Glucose consumption and liver specific functions in terms of albumin and urea synthesis of human hepatocytes were evaluated in the hepatic unit with endothelial cells, and compared to the homotypic culture of only hepatocytes for the whole culture time. Samples collected from the stream leaving the PCL HF membrane bioreactor were stored at −20 °C until being assayed.

Albumin was detected and quantified by immunoenzymatic ELISA method. Samples of 100 µL were incubated with 100 µL of anti-human albumin monoclonal antibody conjugated with horseradish peroxidase (Bethyl Laboratories, Inc., Montgomery, TX, USA) at 4 **°**C overnight, in 96-well plates previously coated with 50 μg/mL chromatographically purified human albumin (Sigma, Milan, Italy). After 4 washes, Tetramethylbenzidine (Sigma Aldrich, Milan, Italy) and H_2_O_2_ were added as detection substrates. The enzymatic reaction was run for 7 min and stopped with 8N H_2_SO_4_. Absorbance was measured at 450 nm using a Multiskan Ex (Thermo Lab Systems, Rodano (MI), Italy).

Urea was detected and quantified by colorimetric urea assay kit QuantiChrom**^™^** (Gentaur, Brussels, Belgium) based on the reaction of urea with a working chromogenic reagent, and absorbance measurements at 520 nm. The glucose consumption was detected by Accu-Chek Active assay (Roche Diagnostics, Monza, Italy).

### 2.6. HPLC Analysis of Diazepam and Metabolites

HPLC was used to analyze the drug biotransformation activity of human hepatic unit cultured in the PCL HF membrane bioreactor and treated with 10 µg/mL of diazepam for the whole culture time. Diazepam and its specific metabolites temazepam, oxazepam, nordiazepam and 4-hydroxy-diazepam were extracted by the samples and detected in HPLC (Agilent Technologies, Cernusco sul Naviglio (MI), Italy) with UV detector set at 236 nm, using a C18-RP Purosphere Star 5 µm, 250 × 4.6 mm column, equipped with a precolumn (Merck KGaA, Darmstadt, Germany) as previously reported [12]. Before the injection, samples were treated with 20% of 4 M NaOH, 1:10 isopropanol, 5:1 ethyl acetate (5:1). After 10 min under gentle rocking and 15 min of centrifugation at 200 g, the ethyl acetate phase was evaporated and dried under vacuum condition and the resulting pellet resuspended in 96 µL mobile phase consisting of 25/35/40 acetonitrile/methanol/0.04%triethylamine pH 7.04, respectively. The sample injection volume was 20 µL. The mobile phase was delivered at 0.8 mL/min and the column was operated at ambient temperature. For all the metabolites calibration curves ranging between 10 ng/mL and 10 µg/mL were previously run.

### 2.7. Statistical Analysis

Statistical analysis was performed using Student’s *t*-test on results expressed as mean ± SD of three experiments per configurations; statistical significance was defined as *p* < 0.05 and *p* < 0.01.

## 3. Results and Discussion

### 3.1. PCL HF Membrane Properties

**Figure 2:**
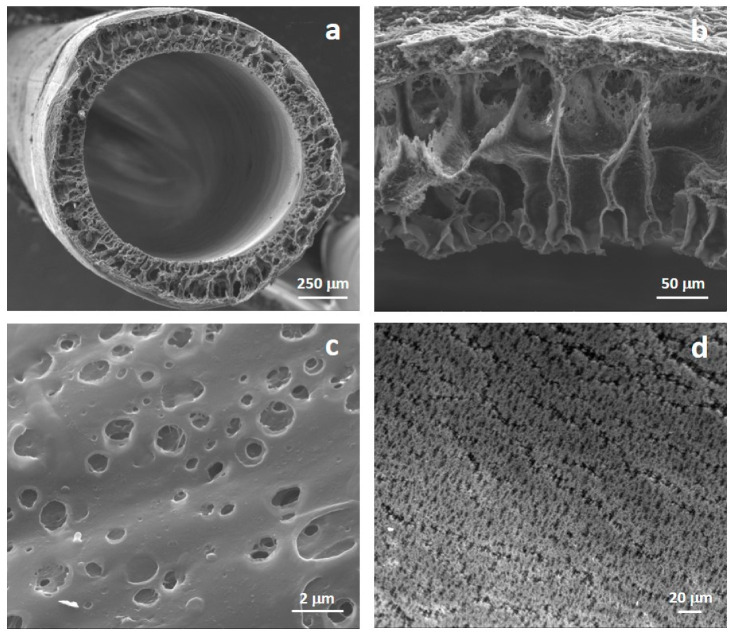
Scanning electron microscopy (SEM) pictures of: (**a**) cross-section, (**b**) wall thickness, (**c**) extracapillary surface, and (**d**) lumen surface of PCL HF membranes.

The morphology of the prepared HFs was analyzed by SEM images that revealed a microporous structure (Figure 2). Fibers show an internal diameter of 1040 ± 90 μm and a wall thickness of 205 ± 40 μm. Microporous interconnected pores are present along the wall of membranes and on both internal and external surfaces. Internal and external membrane surfaces showed pores with mean diameter of 1.3 ± 0.6 μm and 0.4 ± 0.05 μm, respectively.

This morphology is related to the membrane process parameters that were tailored to develop HF microporous structure. The hollow fiber membranes were spun by the dry-jet wet spinning process in which the exchange between solvent and water occurred from the internal side of the hollow fiber membranes when the polymer solution was extruded through the spinneret and when fibers were immersed in the coagulation bath the exchange began from the outside of the membrane. Ultrapure water was employed as bore solution resulting in a rather microporous luminal surface. The bore solution diffused from the internal to the external surface of the HF, which was in contact with air, and the water-NMP exchange occurred with a rate that allow the formation of micropores. Similar pore morphology was observed on the external surface. The fiber geometry and structure including the inner diameter, wall thickness and porosity were established by the process variables such as spinneret geometry, coagulant flow rate, dope extrusion rate, take-up velocity and air gap distance, which were used during the dry-jet wet spinning process.

**Figure 3:**
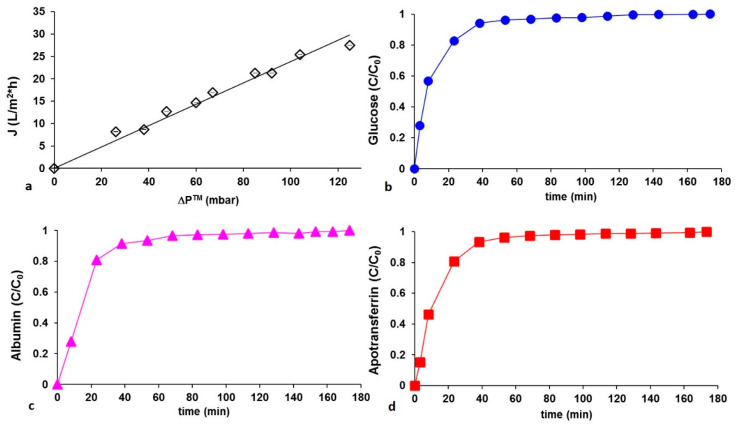
(**a**) Hydraulic permeation measurements of PCL HF membranes at different transmembrane pressures **Δ**P*^™^*. Experimental values were averaged on 10 measurements; the interpolation of experimental data is reported as a solid line: slope (hydraulic permeance): 0.238 L/m^2^·h mbar; R-squared value: 0.98. Concentration appearance profiles of (**b**) glucose, (**c**) albumin, and (**d**) apotransferrin permeating PCL HF membranes at constant transmembrane pressure **Δ**P*^™^* 40 mbar.

The porosity and the wall thickness have an effect on the water permeability. Figure 3a shows the pure water fluxes measurements (J) at different transmembrane pressures (**Δ**P*^™^*) that were used to calculate the hydraulic permeance of the HFs, represented by the slope of the J versus **Δ**P*^™^* straight line. Membranes displayed a hydraulic permeance of 0.238 L/m^2^ h mbar, with an R-squared value of 0.98.

To evaluate the transport properties that play a pivotal role in the communication between hepatocytes and endothelial cells and in the maintenance of cell viability and functions, the permeation of metabolites of interest through the membranes was monitored in the extrafiber space. Metabolites with different chemical properties in terms of MW, Stokes radius and diffusion coefficient in water such as glucose, albumin and apotransferrin were considered. The concentration profiles of the species permeating through membranes at **Δ**P*^™^* of 40 mbar increased with time and reached a plateau as displayed in Figure 3b–d. The same trend was observed at different transmembrane pressure differences. The metabolites concentration on the shell side of the fibers reached stationary values within 45 min demonstrating the fibers capability to ensure their transport.

**Figure 4:**
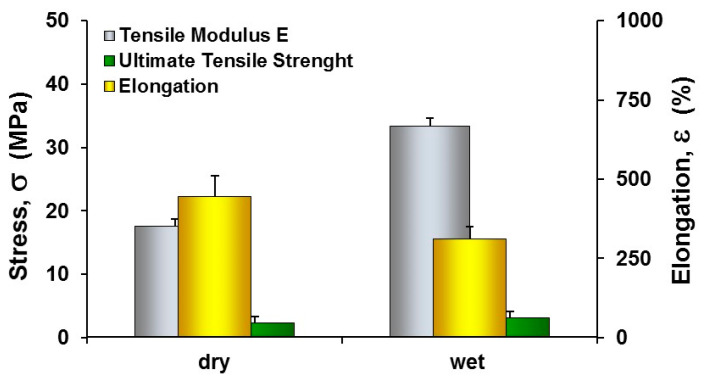
Mechanical properties of PCL HF membranes in dry and wet conditions. The values are the means of 10 measurements per sample ±SD.

Since cells are able to respond to a wide range of external signals offered by the culture substrate, including mechanics of the interface, which leads to morphological and functional changes, we explored the mechanical properties of PCL HF membranes. The results demonstrated that PCL HFs exhibited in dry conditions tensile modulus E and UTS values of 17.6 ± 1.2 MPa and 2.3 ± 0.17 MPa (Figure 4), respectively displaying an extensibility and strength similar to those obtained in other studies by using spinning solution at concentration of 15% [16]. In wet conditions the incorporation of water molecules re-established new hydrogen bonds with the polar functional groups, reducing the elongation at break and increasing the tensile strength and Young’s modulus. The mechanical properties of PCL fibers defined by its elastic modulus can play an important role in regulating the behaviors of cells contributing to the maintenance of tensional homeostasis and normal tissue structure and function.

### 3.2. Cellular Morphology of PCL HF Human Hepatic Unit

**Figure 5:**
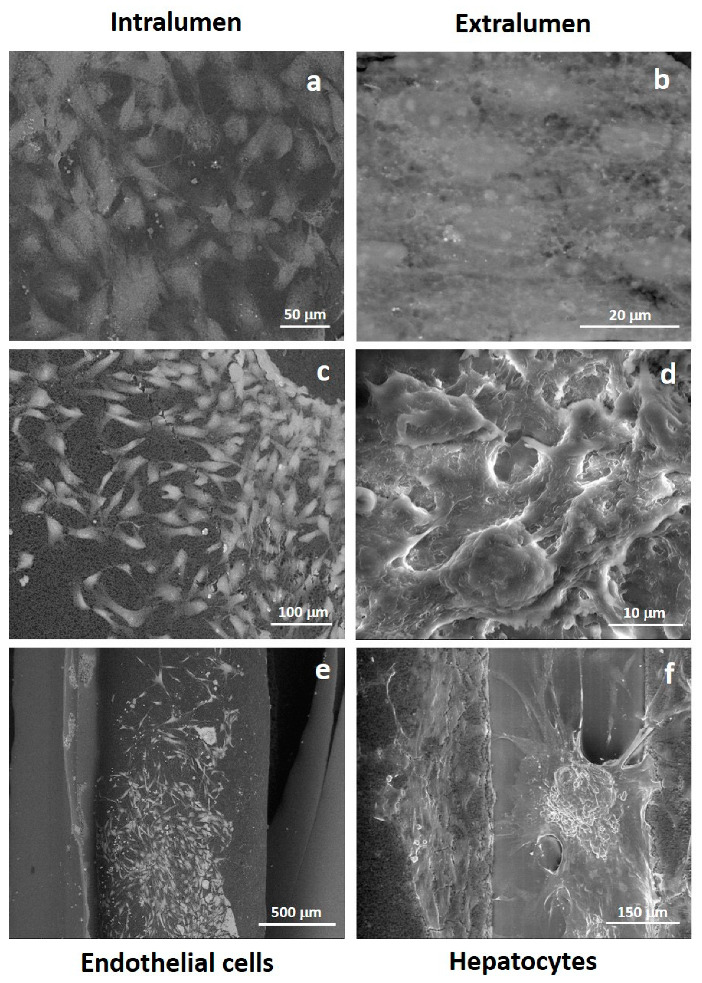
SEM pictures of primary human hepatocytes and human endothelial cells after 18 days of culture in PCL HF membrane bioreactor: (**a**,**c**,**e**) endothelial cells in the intraluminal compartment; (**b**,**d**,**f**) hepatocytes over and between fibers in the extraluminal compartment.

The morphology and adhesion of endothelial cells and hepatocytes seeded in the lumen and shell of the PCL HFs was evaluated after 18 days of culture. Endothelial cells adhered to the lumen surface preserving their specific cobblestone shape and covering completely the internal surface of the fibers. Primary hepatocytes adhered on the external surface of the fibers and established extensive cell-to-cell contacts. They acquired a slightly cuboidal shape forming 3D aggregates into clusters, often with a cordlike appearance reminiscent of hepatic trabeculae (Figure 5).

**Figure 6:**
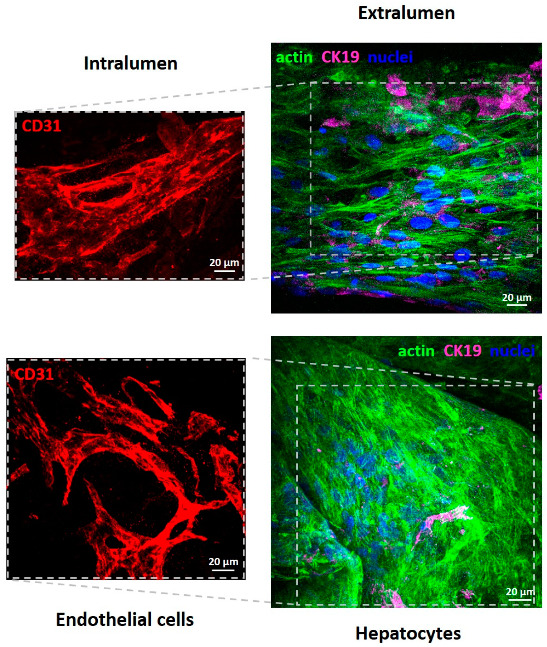
Confocal Laser Scanning Microscopy (CLSM) images of primary human hepatocytes and human endothelial cells after 18 days of culture in PCL HF membrane bioreactor. Endothelial cells in the intraluminal compartment were visualized for CD31 (red); hepatocytes over and between the fibers in the extraluminal compartment were stained for actin (green), CK19 (magenta), and nuclei (blue).

A deeper analysis on the cellular morpho-architecture by CLSM after specific immunostaining, demonstrated that endothelial cells retained the immunoreactivity for CD31, and differentiated in elongated cells that delineate tube- and ring-like structures resembling capillary features (Figure 6). Hepatocytes over the fibers displayed actin organized in microfilaments distributed into the cytoplasm and especially around plasma membrane.

It is interesting to note that cell morphology and tubular structure formation occurred in the lumen surfaces is due, presumably, to the dynamic medium flow (0.5 mL/min) inside the fibers that would generate hydrodynamic shear and enhance the mass transport, which are critically important for ECs. In a previous work we observed the formation of tube-like structures in a flat membrane system by co-culturing human hepatocytes with endothelial cells on membranes with different properties (e.g., porosity, stiffness) [14]. The formation of capillary-like structure was positively influenced by the heterotypic interactions. In this study, endothelial cells are cultured in the lumen of fibers and hepatocytes in the shell of fibers, therefore they communicate by their secreted molecules that permeate through the microporous structure of the HFs membrane wall. It is known that hepatocytes secrete a variety of angiogenic factors (e.g., vascular endothelial growth factor VEGF, transforming growth factor TGFα, acidic and basic fibroblast growth factors aFGF and bFGF, epidermal growth factor EGF, and angioprotein 1) that by permeating through the membranes triggered the angiogenic differentiation of endothelial cells [17]. On the other hand, endothelial cells provide paracrine factors that can affect hepatocyte functions.

### 3.3. Liver Functions of PCL HF Human Hepatic Unit

**Figure 7:**
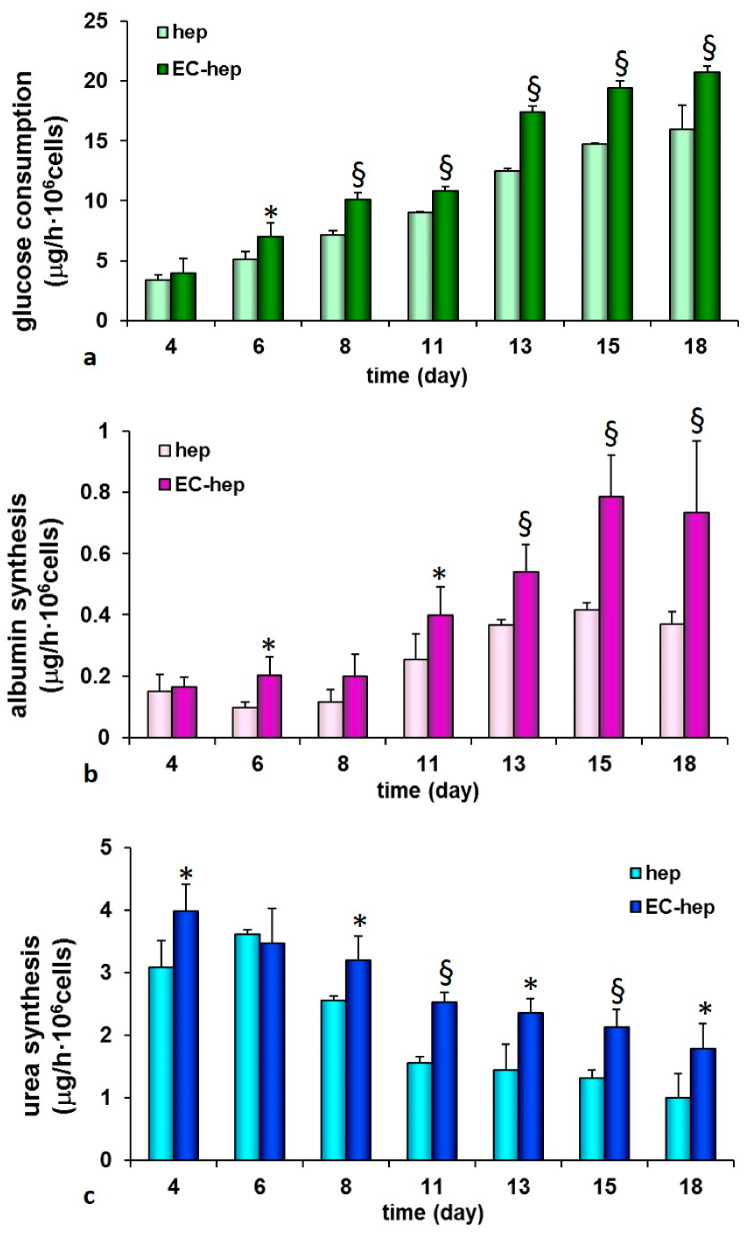
Glucose consumption (**a**), albumin (**b**) and urea (**c**) synthesis by primary human hepatocytes cultured in the PCL HF membrane bioreactor with human endothelial cells (EC-hep) in comparison to homotypic culture of only hepatocytes (hep). The values are the mean ± standard deviations of three experiments per configurations. Data statistically significant according Student *t*-test: (*) *p* < 0.05; (§) *p* < 0.01.

We explored the functional features of cells inside the human hepatic unit by assessing the consumption of glucose since it is the primary source of energy and an important nutrient in sustaining cell growth and viability. Figure 7a shows the increase of cellular glucose uptake with culture time as result of the enhanced metabolic request by hepatocytes and endothelial cells. The glucose uptake was significantly higher in the hepatic unit (17 μg/h·10^6^ cells at day 13) with respect to the monoculture constituted by only hepatocytes. Glucose is important for the energetic metabolism that is required for cell proliferation and functions, as well as for the angiogenic activation that occurred within the fiber lumen. To evaluate the maintenance of liver-specific functions of the bioengineered construct, albumin synthesis and urea production were assessed. A peak of albumin production was reached at day 15 (0.78 ± 0.13 μg/h·10^6^ cells) and then the metabolic rate was maintained up to 18 days of culture (Figure 7b). It is notable that the albumin production of the construct progressively enhanced throughout the period of the experiment. The measured metabolic values were significantly higher with respect to those observed in the control experiment by using only hepatocytes in the same device. The regulation of albumin expression has been shown to be transcriptionally controlled by various transcription factors that could include the one of which seems to cause the increase in albumin expression observed in hepatocytes co-culture [18]. Consistently with the sustained albumin production urea synthesis was maintained for the whole culture time at values ranging from 4 μg/h·10^6^ cells to about 2 μg/h·10^6^ cells. Additionally, urea was synthesized at higher rates in the membrane system in which endothelial cells are cultured inside the lumen of fibers, as a confirmation of the beneficial effect of the paracrine factors secreted by the endothelial cells on hepatocyte functions (Figure 7c).

The presence of endothelial cells increased hepatic albumin (50%) and urea synthesis (44%) at day 18 with respect to the monocultures. The biochemical cross talking of hepatocytes with endothelial cells together with the developed vascularized structures inside the lumen of fibers played a synergic role in the improvement and retention of the liver metabolic functions. The intercellular communication through chemical molecules depends also on the mass transfer properties of the membranes that govern the transport of molecules between the intraluminal and extraluminal compartment, as well as on the fluid dynamics conditions that affect the retention time and homogenous distribution of molecules in the shell compartment.

**Figure 8:**
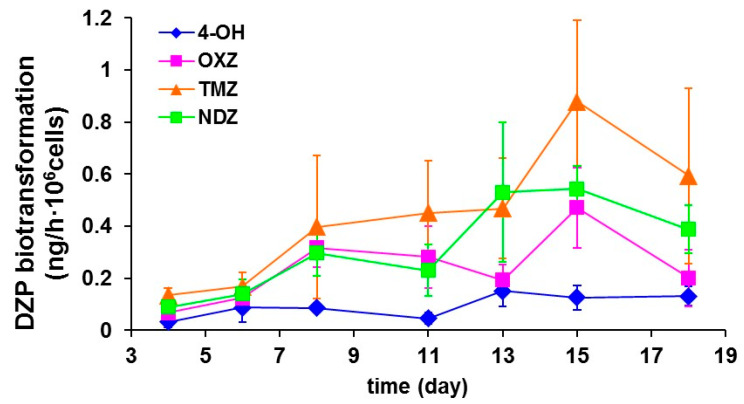
Diazepam biotransformation by primary human hepatocytes cultured in the PCL HF membrane bioreactor with human endothelial cells: temazepam (TMZ), nordiazepam (NDZ), 4-hydroxydiazepam (4-OH) and oxazepam (OXZ). The values are the mean ± standard deviations of three experiments.

The hepatic construct displayed diazepam biotransformation that was retained at high metabolic rate for the whole culture time (Figure 8). In particular, the diazepam elimination rate ranged from 122 to 147 ng/h·10^6^ cells up to 18 days; cells metabolized diazepam through the phase 1 reactions catalyzed by cytochrome P450, producing mainly oxazepam, temazepam, nordiazepam and 4-hydroxy-diazepam. In particular, diazepam N-demethylation is catalyzed by CYP 2B6, 2C9 and to a lesser extent by 3A4 and 3A5. Diazepam 3-hydroxylation activity is catalyzed mostly by CYP3A5, 3A4 and 2C19 [19,20]. The formation of oxazepam occurs through the catalytic activity of isozymes CYP2 B6, 2C8, 2C18, 2C19, 3A4, 3A5 by the 3-hydroxylation of Nordiazepam or N-Demethylation of Temazepam. Diazepam can be directly oxidized producing 4-hydroxy-diazepam. The hepatic unit produced the same metabolites of phase 1 reactions that are found in humans, suggesting that this model is a reliable tool for the prediction of metabolites in xenobiotics.

Perfusion systems by using parenchymal and non-parenchymal cells cultured in 3D structures in hollow fibers [21,22,23], hydrogels microfibers, multiwell plates and microfluidic devices [24,25,26] have been reported. In most of these systems, parenchymal and non-parenchymal cells are co-cultured in direct contact or in connected culture chambers by flow medium using cell lines. Recently, a 3D liver construct has been developed by assembling cell-laden hydrogel microfibers with HepG2 cells densely packed into the core of microfibers, covered with bovine carotid artery endothelial cells [6]. This system enabled the cultivation of the cells for 5 days. Differently from the other systems, our approach is based on the use of PCL HF membranes that are characterized by structural, mechanical and permeability properties suitable to serve as vessel for the development of a vascularized liver construct. The advantage of using HFs in such a system is the ability to fabricate fibers with controlled pore size and mechanical strength in order to create a microenvironment highly selective at molecular level, which is important for the cross talk between cells through paracrine factors secreted by both hepatocytes and endothelial cells. The culture of endothelial cells in the lumen of fibers allowed creating a vascular structure under perfusion that is required for the development of a 3D tissue. In addition, the HF-based hepatic construct may provide immunoisolation and superior mass transport in which high cell densities, continuous flow and scaling capacity can be reached. Cells in the PCL HF hepatic unit tend to be more in vivo-like in morphology and more responsive to experimental cues exhibiting a more polyhedral morphology, enhanced viability, increased albumin and urea synthesis, greater induction of cytochrome-P450 enzyme activity involved in the diazepam metabolism. Furthermore, the use of human cells in our system makes the developed tissue more reliable and the results can be translated to humans.

## 4. Conclusions

In this work, we developed a 3D perfused human hepatic tissue based on biodegradable hollow fiber membranes of poly(ε-caprolactone) (PCL) that compartmentalize human hepatocytes on the external surface and endothelial cells into the lumen of the fibers. The PCL HF membranes displayed permeability, structural and mechanical properties that supported the cell adhesion and functionality. Endothelial cells colonized the lumen of the fibers, forming vascular structures, and communicated with hepatocytes present in the extraluminal compartment through their secreted molecules that permeate across the microporous structure of the membrane wall. The hepatic tissue retained functional activity for 18 days, sustaining glucose consumption, albumin synthesis, urea production and drug biotransformation functions. These results indicated that the developed PCL HF membranes together with the fluid dynamic conditions created a physiologically relevant microenvironment that promoted the biofabrication of vascularized hepatic unit establishing a model that can be applicable to other tissues and organs.
